# Regulatory Challenges in the COVID-19 Era: The Case of Tunisia

**DOI:** 10.1017/dmp.2020.432

**Published:** 2020-11-04

**Authors:** Dora Cherif, Hajer Felfel, Chema Drira, Mariam Aounallah, Meriem Kadri, Mohamed Chiheb Ben Rayana, Myriam Razgallah Khrouf

**Affiliations:** 1 Directorate of Pharmacy and Medicines, Tunis, Tunisia; 2 Faculty of Pharmacy, University of Monastir, Monastir, Tunisia; 3 National medicines Control Laboratory, Tunis, Tunisia; 4 Marrow Bone Transplant Center, Tunis, Tunisia

**Keywords:** public health, health planning organizations, health-care economics and organizations

## Abstract

In view of the possible disruptions in the manufacturing and supply of health products following the coronavirus disease 2019 (COVID-19) pandemic, the Tunisian medicines regulatory authority was mobilized to guarantee patient safety. Teleworking has become the ultimate way of service continuity. The planning was revised according to health priorities. Work procedures were set online. A minimum list of medicines known as "medicines of health and strategic interest" was established. The Directorate of Pharmacy and Medicines (DPM) has been working on updating medicines stock data. A provisional suspension of authorizations for medicines export for 1 mo was decided. A fast-track procedure allowing the validation of alternative sources of raw materials has been put in place. An appeal for a fast track manufacture of hydroalcoholic gel/solutions was launched. A Monitored Emergency Use of Unregistered and Investigational Interventions (MEURI) procedure has been adopted in order to dispense off-label prescriptions of hydroxychloroquine and azithromycin combination. Focus groups were organized in order to set up therapeutic trials exploring possible strategies of COVID-19 treatment, such as serotherapy and BCG vaccine. This proactive and anticipatory policy has made it possible to meet the health challenges dictated by this crisis.

The coronavirus disease 2019 (COVID-19) pandemic is an unprecedented health, economic, and social crisis.^1-5^ In view of the possible disruptions in the manufacturing of health products and supply shortages following the COVID-19 pandemic, the Tunisian medicines regulatory authority was mobilized to ensure the availability of medicines and medical devices and to guarantee patient safety.

The Directorate of Pharmacy and Medicines (DPM), a technical-administrative unit of the Ministry of Health, and the National Medicines Control Laboratory (LNCM) were being challenged for the pandemic. During the period of general containment decreed in Tunisia in early March, DPM and LNCM had to ensure services continuity and react to this public health emergency.

Assessors from DPM and LNCM were forced into containment. Confronted with this unprecedented situation, an active collaboration between the Ministry of Health’s compute center (CIMS), DPM, and LNCM was urgently established to put in place logistical means for a new organization of work. Teleworking has become the ultimate way of service continuity. The planning was revised according to health priorities. Work procedures were set online.

The most important challenge faced by the regulatory authority was the availability of essential medications. Indeed, confronted with the export’s interruption of health products from certain countries, especially China, and the disruption of the supply chain of some pharmaceutical products and raw materials, diplomatic efforts were made to sensitize partner countries on health situation criticality. On the other side, an essential list of medicines known as “medicines of health and strategic interest” was established in partnership with the scientific societies. These medicines were secured through close collaboration between the Central Pharmacy of Tunisia (PCT), the national purchasing office, and DPM.

Concerning locally manufactured medicines to anticipate any stock fluctuations, DPM has been working on updating stock data so as to be able to respond proactively to potential supply problems. The Tunisian pharmaceutical laboratories were informed by means of a press release from DPM of the provisional suspension of authorizations for medicines export for 1 mo, renewable according to the health situation evolution in the country. However, export exemptions have been granted for certain pharmaceutical specialties for which the national stock covers several months to support partner countries.

To respond to shortages of certain raw materials, a fast-track procedure allowing the validation of alternative sources has been put in place. The submission of requests for variations to marketing authorization dossiers relating to the addition of new suppliers of active substances was performed online without prior appointment. These applications are processed on a “fast track” procedure. Forty-one alternative sources of raw materials applications were validated, which represent 4 times the 2019 average.

An appeal to local pharmaceutical manufacturers for the production of hydroalcoholic gel/solutions was launched by the regulatory authority following a simplified and accelerated procedure. Following an ad hoc multidisciplinary committee in partnership with the Faculty of Pharmacy of Monastir, the Order of Pharmacists and representatives of the local industries, 2 formulations were validated and published on the DPM website. To secure the national requirements for alcohol used in the formulation of antiseptics, active coordination with the alcohol authority has been established. Between January and August 2020, a total of 39 compliance decisions on hand sanitizers and surface disinfectants were issued versus 30 in 2019. The accelerated procedure put in place made it possible to reduce the processing time from 3 to 4 mo to around 15 d.

To guarantee the availability of medical devices (MDs) on the Tunisian market, the regulatory authorities have worked in collaboration with the various importers of MDs to evaluate the state of the stock. The logistical support of the customs services made it possible to streamline the MD circuit subject to the temporary withdrawal authorization. At the same time, 525 new consumption authorization applications (CMAs) were submitted online and evaluated on a fast-track process by DPM and LNCM experts.

To guarantee patient’s access to their medicines, a decree-law was promulgated authorizing, for the first time, the renewal and dispensing of electronic prescriptions throughout the period of general confinement. A working group involving the legal unit of the Ministry of Health and ordinary pharmaceutical structures in Tunisia was studying ways of thinking about pharmacies geolocation and the home delivery of health products.

A literature review has been initiated to monitor scientific advances in COVID-19–positive patients’ management. A regulatory framework developed by the WHO and called the Monitored Emergency Use of Unregistered and Investigational Interventions (MEURI) related to hydroxychloroquine procedure has been published on DPM website. This procedure was developed in collaboration with physicians, hospital pharmacists, and pharmacologists to allow clinicians to dispense off-label combined hydroxychloroquine and azithromycin prescriptions in particular clinical situations. During the first wave, approximately 100 COVID-19 patients were treated in accordance with the MEURI procedure.

The marketing authorization applications for locally manufactured generic hydroxychloroquine and all medicines used in COVID-19 patients’ management were prioritized. These applications were submitted online by means of a platform shared between DPM and LNCM and evaluated by remote experts.

To reduce the risk of misuse of chloroquine and hydroxychloroquine, a medical press release issued by DPM was sent to hospital and dispensary pharmacists limiting their dispensing to prescriptions with presentation of an explanatory letter. The goal of this statement was to keep the continuity of care for chronic patients using these molecules and optimize national stock distribution. In the same context, the release calls on wholesale distributors to submit a monthly report on chloroquine and hydroxychloroquine consumption to the regional inspectorate.

Focus groups in collaboration with DPM were organized to set up therapeutic trials exploring possible avenues of treatment, such as serotherapy. Furthermore, clinical experts were encouraged to conduct institutional clinical trials. The procedures for granting authorizations for clinical trials relating to therapeutic strategies to combat severe acute respiratory syndrome coronavirus 2 (SARS-CoV-2) have been streamlined in partnership with the Committee for the Protection of persons.

## Discussion and Conclusions

Regulatory authorities faced a real challenge during the COVID-19 pandemic to ensure patient safety in a health emergency. In Tunisia, the medicines regulatory authority had to adapt quickly to the new measures related to containment. To cope with possible raw materials shortages, Tunisia should put in place a strategy to promote the local active substances industries. A new organization of remote work, digitalization, online procedures have been implemented to ensure services continuity as soon as possible. A proactive policy for securing strategic stocks of medicines, vital MDs, and antiseptics prevented shortages throughout the general containment period. The use of the MEURI procedure allowed managing patients hospitalized with severe forms of COVID-19. During the first wave of COVID-19 in Tunisia, the process ran smoothly and the measures taken by public decision-makers were efficient, despite the emergency situation.
